# Plasma Levels of Occludin and Claudin-5 in Acute Stroke Are Correlated with the Type and Location of Stroke but Not with the Neurological State of Patients—Preliminary Data

**DOI:** 10.3390/brainsci10110831

**Published:** 2020-11-09

**Authors:** Anetta Lasek-Bal, Anna Kokot, Daria Gendosz de Carrillo, Sebastian Student, Katarzyna Pawletko, Aleksandra Krzan, Przemysław Puz, Wiesław Bal, Halina Jędrzejowska-Szypułka

**Affiliations:** 1 Department of Neurology, School of Health Sciences, Medical University of Silesia, 40-055 Katowice, Poland; ppuz@tlen.pl; 2 Department of Neurology, Upper-Silesia Medical Center, Stroke Interventional Therapy Center, 40-635 Katowice, Poland; anikok19@gmail.com (A.K.); aleks.krzan@gmail.com (A.K.); 3 Department of Physiology, School of Medicine, Medical University of Silesia in Katowice, 40-055 Katowice, Poland; dariagendosz@gmail.com (D.G.d.C.); neurologia@gcm.pl (K.P.); hjs13@wp.pl (H.J.-S.); 4 Faculty of Automatic Control, Electronics and Computer Science, Silesian University of Technology, 44-100 Gliwice, Poland; Sebastian.Student@polsl.pl; 5 Department of Outpatient Chemotherapy, Maria Skłodowska-Curie, Memorial National Institute of Oncology, 44-101 Gliwice, Poland; balwieslaw@gmail.com

**Keywords:** stroke, occludin, claudin, zonulin, blood–brain barrier

## Abstract

The blood–brain barrier is the structure (BBB), which isolates the central nervous system from the external environmental. During a stroke, the BBB gets damaged, which is accompanied by changes in the concentrations and distributions of claudin-5, occludin, ZO-1, and other building blocks of the BBB. The aim of this study was to assess the concentrations of selected components of the BBB—occludin, claudin-5, and zonulin (ZO-1)—and to define a potential relationship between the concentrations of these three substances and the type of stroke, the location and extent of the infarct focus, the neurological/functional status in the acute phase of the disease, and the patient’s clinical profile. Methods: In this prospective study, we qualified patients with first-in-life stroke. All patients were analyzed according to: the presence of comorbidities, type of stroke (OCSP), treatment type in the first day of hospitalization, hemorrhagic transformation of infarct focus (ECASS), neurological status on the first day of stroke (NIHSS), functional status (mRS) on the ninth day of disease. In all patients, the plasma concentrations of claudin-5, occludin, and ZO-1 on the first day of stroke were examined and next, the mean concentrations were analyzed and compared between subgroups created on the basis of demographical and clinical features. Results: The mean concentration of occludin was significantly higher in patients with partial anterior cerebral infarct (PACI) compared to patients with posterior cerebral infarct (POCI; 1.03 vs. 0.66 ng/mL; *p* = 0.009) and in patients with location of ischemic stroke in the carotid artery supply compared with in the vertebrobasilar supply (respectively: 1.036 vs. 0.660 ng/mL; *p* = 0.009). The mean concentration of claudin 5 was significantly higher in patients with PACI compared to patients with POCI (0.37 vs. 0.21 ng/mL; *p* = 0.011) and in patients with location of ischemic stroke in the carotid artery supply in comparison with vertebrobasilar supply (respectively: 0.373 vs. 0.249 ng/mL; *p* = 0.011). The differences in mean occludin and claudin 5 concentrations between female and male were statistically not significant, similarly between patients < 65 years and older. A significantly higher mean concentration of zonulin was observed in patients > 65 years of age compared to younger patients (0.59 vs. 0.48 ng/mL; *p* = 0.010) and in patients with arterial hypertension compared to patients without the disease (0.63 ng/mL vs. 0.26 ng/mL; *p* = 0.026). There were no statistically significant relationships between the concentration of occludin, claudin 5, and zonulin and the neurological status according to the NIHSS on the first day of stroke. Conclusions: The location of stroke in the anterior part of the brain’s blood supply is associated with high blood levels of occludin and claudin 5 in the acute phase of stroke. The blood concentration of occludin is significantly lower in lacunar stroke comparing to this in non-lacunar stroke. Old age and arterial hypertension correlate positively with the concentration of zonulin 1 in acute stroke. There is no relationship between the blood levels of occludin, claudin 5, and zonulin 1 on the first day of stroke and the neurological and functional status in the acute phase of the disease.

## 1. Introduction

The anatomical and functional isolation of the central nervous system (CNS) from the external environment is provided by the blood–brain barrier (BBB). It is formed by a group of cells responsible for the selective passage of molecules from the blood into the brain. This is aimed at protecting the CNS from harmful factors and enabling transportation of the compounds circulating in the blood into the cerebrospinal fluid (CSF). In addition to the above roles, the BBB is involved in the efficient maintenance of homeostasis. The BBB between the blood and brain parenchyma is formed by a tight structure of endothelial cells bonded by a dense network of high-resistance junctional adhesion molecules (JAM) including tight junctions (TJs). The latter reveal high electrical resistance and are particularly important in maintaining the tightness of the BBB. The main components of TJs are occludins and claudins, which bond with the cytoskeleton through zonulins while providing additional tightening of the BBB.

The tightness of the BBB during acute stroke becomes dysregulated at an early stage of the disease (at 4–5 h following stroke) [1,2,3,4]. This dysregulation of the BBB results in vasogenic edema and an increased risk of hemorrhagic transformation of the ischemic focus. Thrombolytic therapy (recombinant tissue plasminogen activator, rtPA) applied in stroke increases the risk of hemorrhagic conversion of the ischemic focus in patients whose BBB has broken down but the assessment of the BBB prior to administration of rtPA is highly challenging. We are currently seeking methods to assess the integrity of the BBB so that in the future, we can estimate the risk of post-thrombolysis intracranial hemorrhage in the pretreatment period. In stroke patients, the serum concentrations of occludin and claudin, the key structural components of the BBB, result from damage to the BBB. To date, it has been established in animal and human studies that the levels of occludin and claudin change over time on the initial days following the onset of stroke. Some authors have demonstrated that high levels of occludin remain for more than 24 h after acute brain ischemia and are stable in comparison to the parameters taken at 12 h [5]. It has not been established what determines the plasma concentration of these substances, but stroke type and comorbidities seem to be important. It is known that arterial hypertension is the main cardiovascular risk factor for stroke related to small and large vessel diseases [6]. The presence of hypertension may influence the concentration of BBB damage biomarkers during acute stroke. It is still unknown why the above concentrations reach certain levels in stroke patients. This study was initiated to the answer to this important question.

The aim of this study was to assess the concentrations of selected components of the BBB: occludin, claudin-5, and zonulin (ZO-1), and to define a potential relationship between the concentrations of these three substances and the type of stroke, the location and extent of the infarct focus, the neurological/functional status in the acute phase of the disease, and the patient’s clinical profile.

## 2. Methods

Our prospective study conducted in 2017 included 88 patients with first-in-life stroke, as manifested clinically and identified according to the WHO clinical criteria, confirmed by the result of computed tomography and/or magnetic resonance imaging of the head. The other main inclusion criteria were: the extent of time from the onset of stroke symptoms to hospital admission ≤24 h and pre-stroke status according to modified Rankin Scale (mRS) ≤1 point.

All patients included in the study were analyzed according to:

- Their age at first-in-life stroke;
- The presence of comorbidities, such as atrial fibrillation (AF), arterial hypertension (AH), coronary artery disease (CAD), myocardial infarction (MI) in the last month, diabetes mellitus (DM), lipid disorders (LD), >70% atherosclerotic carotid artery stenosis (CAS, ipsilaterally to the acute ischemic brain lesion);
- Type of stroke using the Oxford Community Stroke Project classification (OCSP) [7];
- Treatment type on the first day of hospitalization (recombinant tissue plasminogen activator (rtPA), thrombectomy, no-reperfusion therapy (antiplatelet);
- Hemorrhagic transformation of infarct focus using the ECASS (European Cooperative Acute Stroke Study) scale [8];
- Their neurological status on the first day of stroke evaluated on the NIHSS (National Institute of Health Stroke Scale) [9];
- Plasma concentrations of the following markers on the first day of stroke: claudin-5, occludin, ZO-1;
- Their functional status on the ninth day following stroke onset according to the mRS scale [10].

The diagnosis of AH was consistent with the recommendations of the European Society of Cardiology (ESC); DM was diagnosed according to the criteria of the Diabetes Association; dyslipidemia was defined according to the ESC recommendations (Guidelines for the Management of Dyslipidemias) [11,12,13].

The degrees of stenosis of the common carotid artery and/or internal carotid artery were assessed according to the NASCET criteria [14].

Markers assessment was performed by serum isolation. Serum was obtained by venipuncture. According to collection protocol, blood samples were left at room temperature to allow clotting for 60 min. T serum was isolated by centrifuging the blood sample at 1400× *g* for 15 min at room temperature, after which the supernatant was pipetted carefully into aliquots that were immediately stored at −80 °C. Hemolytic samples were excluded from the analysis.

Measurement of blood biomarkers was carried out as such; targeted proteins were measured with commercially available enzyme-linked immunosorbent assay (ELISA) kits according to the manufacturer’s protocol (zonulin-1, EIAab, China; occludin, EIAab, China; claudin-5, Fine Test, China): 100 µL for zonulin-1, 100 µL for occludin, 100 µL for claudin-5. The mean value of the serum concentrations of occludin, claudin-5, and ZO1 was assessed for all patients and divided according to sex. Next, the mean value was assessed in subgroups created according to the presence or absence of the diseases mentioned above and these mean values were then compared between suitable subgroups.

Patients were categorized into four subgroups based on OCSP classification. In each of these subgroups, mean concentrations of the examined substances were assessed and comparisons were made between them. Similarly, the mean concentration of mentioned biomarkers was compared between patients with lacunar versus non-lacunar stroke.

The subgroups were formed according to the treatment type. The mean concentrations of the examined substances were assessed and comparisons were made between them.

Patients were categorized into three subgroups based on their NIHSS score (≤4, 5–12, and >12) assessed on the first day of hospitalization. Comparisons were made between the mentioned subgroups.

Patients were categorized into two subgroups based on their mRS score (≤2 and 3–6) assessed on the ninth day of hospitalization. In each of these groups, mean concentrations of the examined substances were assessed and comparisons were made between the subgroups.

Univariate analyses for differences between male and female groups for binary variables were performed using Pearson’s Chi-squared test. Continuous variables, in particular zonulin, claudin, and occludin, were compared between categorical variables using the Kruskal–Wallis test. A multivariable model was built by using logistic regression and binomial GLM. The model’s variable selection procedures included automatic selection (stepwise, forward, and backward) based on AIC and BIC criterion. For evaluating the accuracy of the predictions made by the model, the “leave one out” procedure and the area under the ROC curves (AUC) or multiclass AUC estimator were used. All statistical analyses were performed using R version 3.6.1. The discriminative power of this model was evaluated by receiver operating characteristic (ROC) area under the curve (AUC) analysis. A non-adjusted cutoff value for adequate sensitivity and specificity was also used.

The study was accepted by the Ethics Committee. All subjects gave their informed consent for inclusion before they participated in the study. The study was conducted in accordance with the Declaration of Helsinki, and the protocol was approved by the Ethics Committee of the Medical University of Silesia in Katowice, Poland of KNW/022/KB1/33/I/14.

## 3. Results

The mean age in whole study group was 73.11 years. The mean age among women was significantly higher than in men (74.91 ± 12.04 vs. 69.08± 9.54; *p* = 0.02). There were no statistically significant differences associated with gender as a result of neurological status (med. 4 points in female vs. med. 3 points in male, *p* = 0.63) and functional status (2 vs. 2; *p* = 0.70) in the acute phase of stroke. The characteristics of our patients are presented in Table 1.

The mean concentrations of occludin, claudin 5, and ZO-1 in the whole group of patients were as follows: 0.923 ± 0.76, 0.339 ± 0.38, and 0.567 ± 0.91 ng/mL, respectively.

**Figure 1:**
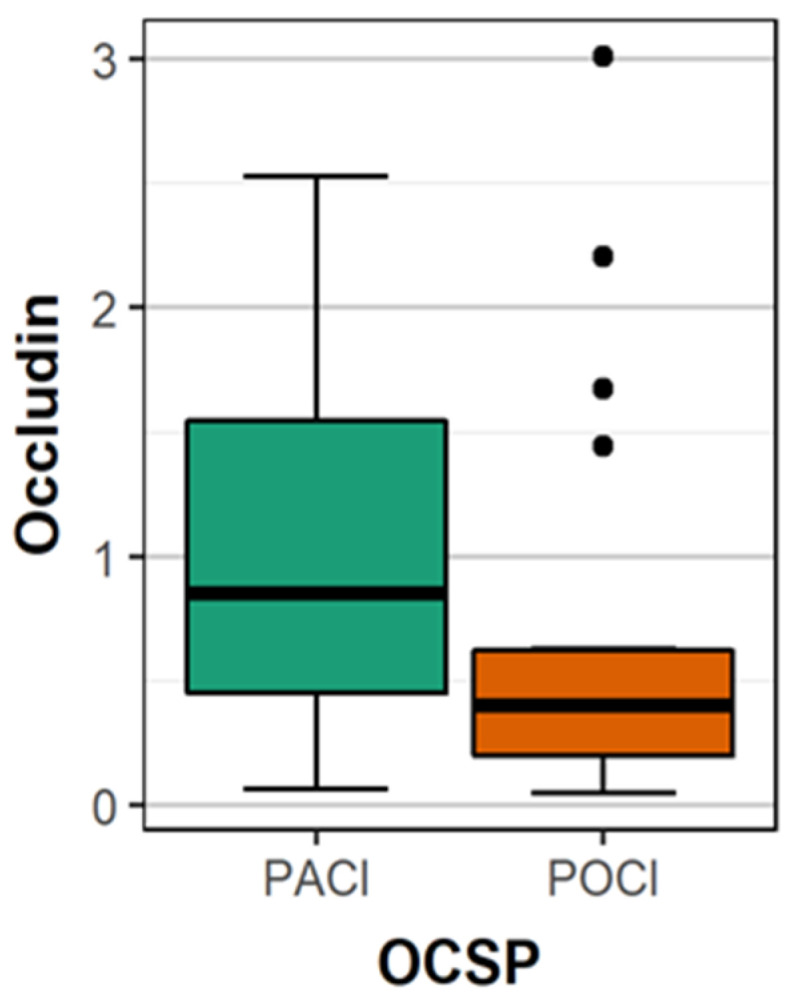
The comparison of the mean concentration of occludin between patients with PACI and POCI.

The mean concentration of occludin was significantly higher in patients with PACI (partial anterior cerebral infarct) compared to patients with POCI (posterior cerebral infarct) (1.03 vs. 0.66 ng/mL; *p* = 0.009) (Figure 1). There were no statistically significant differences between the mean concentration of occludin in patients with other types of stroke. The mean concentration of occludin in patients with location of stroke in the carotid artery supply was significantly more often than in the vertebrobasilar supply. (Table 2) The differences in mean occludin concentrations between female (0.865 ng/mL) and male (1.012 ng/mL) were statistically not significant (*p* = 0.35), similarly between patients <65 years (1.005 ng/mL) and older (0.906 ng/mL; *p* = 0.68).

**Figure 2:**
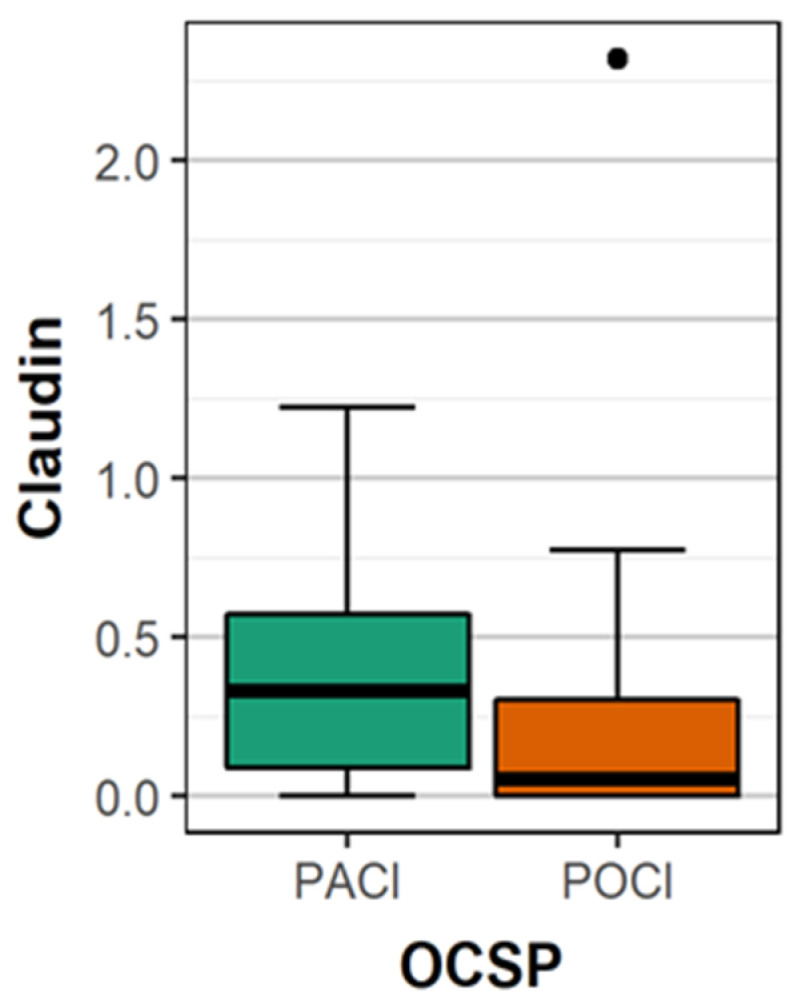
The comparison of the mean concentration of claudin 5 between patients with PACI and POCI.

The mean concentration of claudin 5 was significantly higher in patients with PACI compared to patients with POCI (0.37 vs. 0.21 ng/mL; *p* = 0.011) (Figure 2). There were no statistically significant differences between the concentration of occludin in patients with other types of stroke. The mean concentration of occludin in patients with location of stroke in the carotid artery supply was significantly more often than in the vertebrobasilar supply (Table 2). The differences in mean claudin 5 concentrations between female (0.354 ng/mL) and male (0.322 ng/mL) were statistically not significant (*p* = 0.839), similarly between patients <65 years (0.318 ng/mL) and older (0.346 ng/mL; *p* = 0.878).

**Figure 3:**
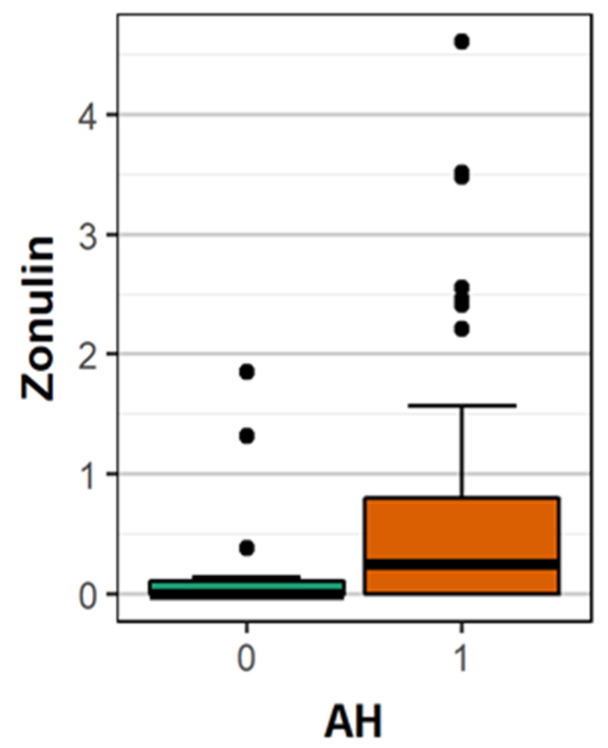
The comparison of the mean concentration of ZO-1 between patients with- and without arterial hypertension.

A significantly higher mean concentration of ZO-1 was observed in patients >65 years of age compared to younger patients (0.59 vs. 0.48 ng/mL; *p* = 0.010) and in patients with arterial hypertension compared to patients without the disease (0.63 vs. 0.26 ng/mL; *p* = 0.026) (Figure 3). There were no statistically significant differences between the concentration of ZO-1 according to the type of stroke (OCSP classification) and gender (female 4.860 ng/mL, male 5.351 ng/mL; *p* = 0.633). Similarly, the differences in location in artery supply were not statistically significant. (Table 2)

There were no significant differences in the mean concentrations of occludin, claudin 5, and ZO-1 between patients with diabetes mellitus, lipid disorders, atrial fibrillation, myocardial infarction, coronary heart disease, and ipsilateral internal carotid artery stenosis (>70%) and not suffering from the above diseases. No significant differences were observed in the mean concentration of occludin and claudin 5 depending on the presence or absence of arterial hypertension.

A significantly lower mean concentration of occludin was observed in patients with lacunar stroke compared to those with non-lacunar stroke (Table 3). There were no significant differences in mean concentration of the other mentioned biomarkers in lacunar versus non-lacunar stroke patients.

There were no statistically significant relationships between the concentration of occludin, claudin 5, and ZO-1 and the neurological status according to the NIHSS on the first day of stroke. (Table 4).

There were no statistically significant relationships between the concentration of occludin, claudin 5, and ZO-1 and the functional status according to the modified Rankin scale on the ninth day following stroke. (Table 5).

Similarly, no statistically significant correlation was found between the mean concentration of occludin, claudin, and ZO-1 and the type of therapy on the first day of stroke.

The independent factors of a favorable prognosis (mRS 0–2) were identified as: NIHSS <4 on the first day of stroke, no AF- and CAD-burden, and age ≤65 years.

The independent factors of a poor prognosis (mRS 3–6) were identified as: age >65 years. Only two patients experienced hemorrhagic transformation of ischemic focus—PH2 according to ECASS. The number of patients was too low to compare with the others.

## 4. Discussion

The most important result of the study was the finding that concentrations of occludin and claudin are associated with the type of stroke and stroke location. ZO-1 concentration is not related with the type or anatomical location of stroke; it is related with age and the presence of arterial hypertension. There is no correlation between claudin-5, occludin, or ZO-1 blood concentrations on the first day of stroke and the neurological and functional status of patients in the acute disease period. In respect of the above results, the substances under study can probably be the markers of the extent of the morphological changes to the central nervous system; they reflect the degradation of the BBB, most likely as a result of reperfusion injury. This is an important clinical finding and one that is consistent with the results of animal studies [5]. This means that the assessment of plasma occludin and claudin concentrations in the pre-treatment period could help us estimate the risk of hemorrhagic conversion of the infarct focus. The biochemical factors that would improve the stratification of the risks of an unfavorable evolution of the infarct focus and the complications following rtPA application are being investigated. Claudin and occludin occur in the blood of patients during the acute period of stroke as a result of the dysregulation of the blood–brain barrier. The loss of BBB integrity results from acute ischemia and the damage caused by reperfusion.

During a stroke, the BBB gets damaged, which is accompanied by changes in the concentrations and distributions of claudin-5, occludin, ZO-1, and other building blocks of the BBB [1,2]. The primary role of claudins is to ensure BBB tightness and to control paracellular permeation [15,16]. During stroke, an altered expression of mRNA was observed along with a decrease for several classes (claudins 1,3,12) and overexpression for claudins 5,11,25 [17]. Additionally, differences in claudins concentrations were found depending on the time of stroke, with their peaks on day one and three [2]. Occludin is a critical transmembrane regulator of BBB functional integrity [18,19]. It plays a key role in the response to redox change and acts as a redox sensor and regulator at the TJ [20,21]. It participates in the signal transducing of cytokines such as TNF-alpha and interferon gamma [22]. JAM isoforms regulate paracellular permeability at the BBB, particularly with respect to immune cells (i.e., neutrophils, monocytes/macrophages) [23,24]. A loss of JAM protein expression and/or migration of JAM proteins away from the tight junction directly lead to a loss of BBB properties at the microvascular endothelium [25,26]. Previous studies have shown that the dissociation of ZO-1 from the junction complex is associated with increased permeability, indicating that the interaction of the ZO-1-transmembrane protein with the junction complex is critical for tight junction stability and function [27]. The expression of occludin in the endothelial cells of the brain capillaries is lowered during stroke [28,29]. Occludin is redistributed into cytosol and the paracellular barrier is weakened [21,30]. The presence of claudin-5 and occludin in the blood of patients in the acute stroke period indicates that the BBB has been destroyed and, according to the results of our study, the concentrations of both substances in the blood are positively correlated with the extent of cerebral stroke. In our study, the concentrations of claudin 5 and occludin were higher in patients whose stroke was located in the area of carotid artery vascular supply, as compared with those whose stroke was located in the area supplying the basilar artery. This is most probably related to the extent of damage incurred, which is usually significant in the case of stroke in the anterior part of cerebral circulation. Moreover, the mean concentration of occludin was lower in patients with lacunar stroke. This subtype of stroke is associated usually with a positive outcome [31]. The results of animal studies revealed that 4 h after brain ischemia onset, the concentration of occludin rapidly increases [5]. This may suggest that the release of claudin into the plasma occurs after BBB damage has reached a certain threshold. Experimental studies found that the level of blood occludin concentration results from the extent of BBB damage [5].

The question is: what is the source of occludin and claudin? Does it originate in the area of the penumbra or from the ischemic core? Considering that we measured the parameters before the end of the first day of stroke, the occurrence of occludin and claudin-5 was most probably due to ischemic reperfusion injury. Other authors have observed a bimodal increase in the concentration of the above substances on day 1 and 3 of stroke. It seems that the concentration peaks reflect various stages of the disease: BBB damage due to rapid reperfusion (hyperemia), and on day 3, due to the inflammatory reaction in the infarct focus [2]. Animal studies have demonstrated that high levels of occludin remain for more than 24 h after acute brain ischemia and are stable in comparison to the parameters taken at 12 h [5]. At both time-points, the concentrations were significantly higher than those found before the occlusion of the middle cerebral artery.

According to the results of the presented study, ZO-1 concentration was only positively correlated with the age of patients and the presence of arterial hypertension. So far, experimental animal studies have shown a negative effect of arterial hypertension on the distribution and function of ZO-1 and occludin, which we have also found in relation to ZO-1 in our patients [32]. Based on quantitative radiological methods, it has been shown that older age was associated with a more extensive BBB damage [33]. Our experiments are consistent with the ones published solely in terms of the increase in ZO-1 concentration.

Only six patients were confirmed by head CT on the second day of the disease to have the hemorrhagic transformation of stroke at PH2 level. This may be due to the low percentage of patients undergoing reperfusion therapy; we collected the study material before thrombectomy was included in everyday clinical practice. According to the results of other authors, the blood concentration of occludin and claudin correlated with the risk of hemorrhagic conversion of cerebral infarct [34].

It is now established that the tight junction protein complexes are dynamic in nature and can be organized and reorganized in response to ischemic stroke. Describing the specific role of molecules in BBB damage may prove to be of significant importance for future therapeutic strategies in reducing ischemia reperfusion damage. The substances which increase the expression of claudin-5 have been shown to increase transendothelial electrical resistance and decrease BBB permeability [35]. We suspect that the tight junction can be targeted pharmacologically during ischemic stroke for the purpose of reducing injury and BBB solute leak. In this respect, it is important to establish the reasons on which claudin concentration depends and the changes occurring in the levels of occludin and claudin in the blood of patients during the acute period of the disease.

Limitations: The authors are aware of the limitations of this study, the most important being the relatively low number of patients; however, at this stage of a multi-stage scientific project, the paper is just a preliminary report.

## 5. Conclusions

The location of stroke in the anterior part of the brain blood supply is associated with high blood levels of occludin and claudin 5 in the acute phase of stroke.

The blood concentration of occludin is significantly low in lacunar stroke.

Old age and arterial hypertension correlate positively with the concentration of zonulin 1 in acute stroke.

There is no relationship between the blood levels of occludin, claudin 5, and zonulin 1 on the first day of stroke and the neurological and functional status in the acute phase of the disease.

## Figures and Tables

**Table 1:** Characteristics of the patients.

Parameter	Group *n* = 88
Age (years), mean ± SD [min, max]	73.11 ± 11.48
	[36–103]
Sex, F (*n*)	42 (47.72%)
DM, *n* (%)	22 (25%)
AH, *n* (%)	68 (77.27%)
AF, *n* (%)	31 (35.22%)
MI, *n* (%)	6 (6.81%)
CAD, *n* (%)	29 (32.95%)
CAS, *n* (%)	11 (12.5%)
LD, *n* (%)	55 (62.5%)
Location of stroke, *n* (%)	
TACI	37 (42.04%)
PACI	22 (25%)
LACI	24 (27.27%)
POCI	5 (5.68%)
NIHSS, median (IQR)	3 (5)
[min, max]	[0–29]
Therapy, *n* (%)	
rtPA	22 (25%)
MT	8 (9.09%)
no reperfusion therapy	58 (65.9%)
ECASS classification,	
*n* (%)	
HI1	4 (4.54)
PH2	2 (2.27)
mRS, median (IQR)	2 (4)
[min, max]	[0–6]

*n*—the number of patients, F—female; NIHSS—National Institutes of Health Stroke Scale; DM—diabetes mellitus; AH—arterial hypertension; AF—atrial fibrillation; MI—past myocardial infarct; CAD—coronary artery disease; CAS—carotid artery stenosis; LD—lipid disorder; PACI—partial anterior cerebral infarct; TACI—total anterior cerebral infarct; POCI—posterior cerebral infarct; LACI—lacunar cerebral infarct; rtPA—recombined tissue plasminogen activator; MT—mechanical thrombectomy; ECASS—European Cooperative Acute Stroke Study; HI1—hemorrhagic infarction type 1, PH2—parenchymal hematoma type 2, mRS—modified Rankin Scale.

**Table 2:** The mean concentrations of occludin, claudin 5, and ZO-1 associated with the location of the ischemic focus in the artery supply.

Parameter	ICA Supply	VA Supply	*p*
occludin (ng/mL)	1.036	0.660	0.009
claudin 5 (ng/mL)	0.373	0.249	0.011
zonulin (ng/mL)	0.693	0.236	0.105

ICA—internal carotid artery; VA—vertebral artery, *p*—the value of statistical significance.

**Table 3:** The mean concentrations of occludin, claudin 5, and ZO-1 associated with lacunar vs. non-lacunar stroke.

Parameter	lacunar Stroke	Non-Lacunar Stroke	*p*
occludin (ng/ml)	0.708	1.028	0.026
claudin 5 (ng/ml)	0.293	0.359	0.128
zonulin (ng/ml)	0.249	0.704	0.102

*p*—the value of statistical significance.

**Table 4:** The comparison of mean concentrations of occludin, claudin 5, and ZO-1 in subgroups of patients according to NIHSS (0–4 points vs. 5–12 vs. >12 points).

Parameter/ NIHSS	NIHSS (A) 0–4 *n* = 48	NIHSS (B) 5–12 *n* = 20	NIHSS (C) >12 *n* = 20	NIHSS A vs. B; *p*	NIHSS B vs. C; *p*	NIHSS A vs. C; *p*
occludin (ng/mL)	1.002	0.717	0.955	0.261	0.580	0.909
claudin 5 (ng/mL)	0.378	0.265	0.283	0.625	0.903	0.625
zonulin (ng/mL)	0.492	0.596	0.875	0.904	0.914	0.939

*n*—the number of patients, *p*—the value of statistical significance.

**Table 5:** The comparison of mean concentrations of occludin, claudin 5, and ZO-1 in subgroups of patients according to mRS (0–2 points vs. 3–6).

Parameter/mRS	mRS 0–2 *n* = 51	mRS 3–6 *n* = 37	*p*
occludin (ng/mL)	0.984	0.799	0.503
claudin 5 (ng/mL)	0.304	0.454	0.618
Zonulin (ng/mL)	0.404	0.812	0.183

*n*—the number of patients, *p*—the value of statistical significance.
